# Exploring the reasons why women prefer to give birth at home in rural northern Ghana: a qualitative study

**DOI:** 10.1186/s12884-020-03198-y

**Published:** 2020-08-28

**Authors:** Peter Adatara, Johanita Strumpher, Esmeralda Ricks

**Affiliations:** 1 Department of Nursing, University of Health and Allied Sciences, PMB, 31 Ho, Ghana; 2 Department of Nursing, Nelson Mandela University, P O Box X77000, Port Elizabeth, 6013 South Africa

**Keywords:** Reasons, Women preference, Home birth, Rural northern, Ghana

## Abstract

**Background:**

Skilled birth care during childbirth is reported in the literature as one critical strategy for reducing maternal morbidity and mortality. Despite the importance of birth care provided by skilled birth attendants, women in rural areas of northern Ghana still give birth utilising the birth services provided by Traditional Birth Attendants. The aim of this study,therefore, was to explore and describe the reasons why a small group of rural women chose homebirth in rural northern Ghana.

**Methods:**

A qualitative approach was adopted to explore the reasons why women prefer to deliver at home in rural areas of northern Ghana. Individual interviews were used to obtain a full description of factors and experiences of women associated with home births in rural areas in Ghana. The research population consisted of 10 women who utilised birth care services provided by Traditional Birth Attendants in a rural community of northern Ghana. Data collected from the interviews were transcribed verbatim and analysed to identify themes.

**Results:**

This study, which was conducted among a small group of women, yielded interesting results on why these women still give birth at home in rural northern Ghana. It was found out that perceived poor quality of care and conduct of skilled birth attendants; the perception that women received better care from Traditional Birth Attendants; financial constraints and lack of access to healthcare facilities in the rural areas by these women accounted for majority of the reasons why women in rural northern Ghana still give birth at home.

**Conclusion:**

The study highlighted some barriers experienced by participants to the utilisation of birth care services provided by skilled birth attendants in rural northern Ghana. Management of healthcare facilities should facilitate the implementation of supportive supervision in the maternity units to improve the quality of care and attitude delivered by skilled birth attendants in maternity care in rural communities.

## Background

Skilled birth care during childbirth play an important role in reducing maternal morbidity and mortality [1]. Skilled birth care describes the type of care by which a woman is provided with adequate care during labour, delivery and the immediate postpartum period by a trained health care professional [1]. Skilled care at birth occurs in a health facility or homebirth setting, assisted by trained professionals, including midwives. Evidence reported that about 15% of pregnant women, particularly in developing countries such as Ghana, develop some form of obstetric complications during pregnancy and childbirth which in some cases, result in maternal death [2, 3]. The World Health ‘Organisation’s (WHO) report indicated that globally in 2015, an estimated 303,000 women died due to obstetric complications [4]. While almost of the global maternal deaths occurred in developing countries, about two-thirds of these deaths took place in sub-Saharan Africa [5]. Though the occurrence of obstetric complications sometimes is often unpredictable [6], there exist evidence that ensuring skilled birth attendance during childbirth plays an important role in reducing obstetric complications and maternal deaths [7].

The World Health Organisation (WHO) associates inadequate utilisation of skilled birth care provided by skilled birth attendants such as midwives and doctors in Sub-Saharan Africa as a major hindrance to efforts aimed at improving the health of women, especially during childbirth [8]. Skilled birth care provided by skilled birth attendants during childbirth is one of the key indicators for reflecting progress towards the achievement of the Sustainable Development Goals [1].

Despite the importance of birth care provided by skilled birth attendants, women in rural areas of northern Ghana still give birth utilising the services of Traditional Birth Attendants. The World Health Organisation (WHO) defines a traditional birth attendant as “a person who assists the mother during childbirth and initially acquired her skills by delivering babies herself or through apprenticeship to other traditional birth attendants” [9]. Traditional Birth Attendants (TBAs) continue to play a significant role in assisting childbirth care services, particularly in rural areas of Ghana [10]. The recent maternal health statistics from Ghana Health Service 2016 report shows that more than 80% of pregnant women had at least one contact with a skilled provider during pregnancy and only 56.2% of deliveries were attended by skilled birth attendants [11]. This implies that more than 40% of women still give birth at home, utilising the services of Traditional Birth Attendants (TBAs). This inadequate utilisation of birth care provided by skilled birth attendants makes it difficult to achieve the national target of 80% unless strategies are put in place to motivate women to utilise skilled birth attendants.

Previous studies have shown that in Ghana, women, traditionally prefer to deliver at home because it is cheaper, and easier as women who deliver at home receive social support from their extended families and do not have to pay much for the delivery services [10, 12, 13]. Other studies [14–17] indicate that lack of financial or economic resources, transportation, and delivery of supplies, lack of coordination and referral between Traditional Birth Attendants at the community level and facilities can all inhibit women from using facility-based services. However, most of these studies have been urban-focused, and consequently, rural ‘women’s perspectives have been less discussed or studied. Besides, most of the studies have adopted quantitative approaches which limit a deeper understanding of the factors associated with home births in rural northern Ghana where there is underutilisation of skilled birth care services.

The aim of this study, therefore, is to explore and describe the factors and experiences of a small group of rural women choosing homebirth in rural northern Ghana.

## Methods

The aim of this study, therefore, is to explore and describe the factors and experiences of a small group of rural women choosing homebirth in rural northern Ghana.

### Research design

A qualitative explorative and descriptive research approaches were used to gain an understanding of the reasons accounting for home birth in rural areas of northern Ghana. This design enabled the researchers to explore and understand rural women’s reasons for utilising unskilled birth services provided by Traditional Birth Attendants in rural northern Ghana [18].

### Study setting

The research was carried out in a small District in the Upper East Region of Ghana. The District is one of most rural and deprived districts in Ghana which has all the characteristics of a typical rural area in Ghana [18]. The District has 94 % (94%) of its population residing in rural areas. Also, the District was chosen because it recorded low utilisation of skilled birth care provided by skilled birth attendants at the time of data collection.

The District has one district hospital in the district capital with four reproductive health clinics, and seven completed Community Health-based Planning Services (CHPS) compounds, sixty-two outreach points, ten feeding centres and one rehabilitation centre. Records from the District Health Directorate, showed there was only one medical doctor and sixty-five nurses in the entire district [19]. Midwives mostly provide primary maternity care for women during pregnancy and childbirth. It is imperative to mention that women in the district are exposed to a variety of alternative childbirth sources. Among these include TBAs, traditional healers and herbalists, spiritual healers and diviners.

### Research population

The purpose of this study was to understand why women give birth at home by utilising birth services provided by Traditional Birth Attendants in rural northern Ghana. The research population in this study comprised of women who gave birth using birth care provided by Traditional Birth Attendants in the rural areas in the Bongo District of Ghana.

### Inclusion criteria

To qualify to participate in this study, a participant should be a:

- Mothers who gave birth to live babies at home assisted by traditional birth attendants or family relatives within the past six ‘months’ period.
- Mothers who were above eighteen years at the time of data collection

### Exclusion criteria

This study did not consider participants who fell within the under listed criteria:

- Mothers who gave birth to live babies in health facilities assisted by skilled attendants within the past six ‘months’ period and whose babies at the time of the study were still alive and well.
- Mothers who were below eighteen years at the time of data collection
- Mothers who were sick or whose children were sick at the time of data collection

### Sampling strategy and sample size

A purposive sampling technique was used to select ten (10) participants for individual semi-structured interviews. The sample size of 10 women was based on data saturation [20]. In qualitative inquiry, the sample size is determined based on informational needs. The guiding principle, therefore, is data saturation, that is sampling to the point at which no new information is obtained, and redundancy is achieved [20]. Ten interviews were conducted, and saturation of the data occurred at the 10th participant. The researcher selected participants based on who could give the most and the best information about the objectives of the study. Bongo District is sub-divided into six sub-districts or zones according to the Bongo District Health Directorate. Two Zones were used for the study. The two sub-districts were purposefully selected for the study because of the rural nature of these communities. The researchers contacted nurses and Community Key Informants (CKIs) who provided them with a list of potential participants (women) who delivered at home within six months and also utilised birth care provided by Traditional Birth Attendants in each of the selected zones. Also, only women who were willing to participate in the study and also met the inclusion criteria were recruited for the study. The purpose of the study was then explained to them in order to help them to appreciate what was required of them.

### Data collection

Data were collected through semi-structured interviews using a flexible interview guide to explore to the reasons why women prefer to deliver at home in rural areas of northern Ghana. Thus, participants had the opportunity to tell their story with minimal interruption. The interviews took place in safe, quiet, comfortable, private and mutually agreed-upon locations. The individual interviews were initially planned to take place at the health facilities in the district, but after interviews with two of the participants, it was realised that the participants were distracted by clients accessing maternal health care services in the facilities of the participants. Some of the participants suggested their home as the most preferred venues for the individuals because those venues were free from interruptions. All the participants who agreed to take part in the research were asked to sign an informed consent after reading and receiving information about what was involved in the study. The consent form was read in the Grune, a language spoken in the study area to participants who could not read or write. Such participants were made to thumbprint the consent forms. Interviews were conducted in a Grune language, preferred by participants. The interviews lasted between 45 and 60 min and were recorded. The same questions were posed to all participants (*See Supplementary file* *1*). The audio-taped interviews were transcribed within 24 h of the interview. A language expert translated the interviews into the English language to enable the researchers of the study to understand the content of the interviews.

### Data analysis

Data analysis occurred concurrently with data collection. Our goals were to condense raw data and provide a detailed and thick description of the phenomenon of interest. Data collected through the semi-structured interviews were transcribed verbatim and analysed according.

to the six-step guide proposed by Braun & Clark e[20]. The first step was reading the transcripts to become familiar with the data. The transcripts were read many times while taking down notes and were reviewed independently by two researchers with rich experience in qualitative research for accuracy and to ensure objectivity. The next step was that the data were coded using the NVivo version 12 software and initial codes were generated from the coded data. The coding was done according to the themes of the research questions of this study. After generating many codes, we searched for themes and sub-themes which relevant to the research questions. Codes were put together into themes. Initially, we identified six themes. The identified themes were later merged into four main themes after the reviewed of themes by two of the researchers.

## Results

### Identified themes

This study identified four main themes:

- Women chose to deliver at home because of poor quality of care and conduct of skilled birth attendants
- Women preferred home delivery because Traditional Birth Attendants gave better care;
- Women delivered at home due to financial constraints
- Women lacked access to healthcare facilities

#### Theme 1: women chose to deliver at home because of poor quality of care and conduct of skilled birth attendants

The participants who delivered at home revealed that though they had wanted to deliver at a health facility, they faced insurmountable barriers to utilise facility-based childbirth., As a result of these barriers, women often had little or no option but to deliver at home. One of the reasons accounting for home birth according to the participants was the poor attitudes of skilled birth attendants and poor quality of skilled birth care at the health care facilities.

*"Hmm... (smiling and nodding the head). I must be honest, one of the things that actually made me to deliver at home was the fact that when you go to deliver at the health facility, the nurses would just be insulting you and embarrassing you like that and your colleague pregnant women would just be laughing at you as if you have committed a great crime. So, I 'didn't want to go and deliver in a health facility so that the nurses would insult and laugh at me again" ( IDI,gravida 1 Para 1 woman).*

The participants reported that some of the skilled birth attendants exhibited poor conduct such as using harsh words on them during previous childbirth and so ‘didn’t want to go through a similar experience in their recent childbirth as one of the participants indicated below:

*"As for me, I delivered at home because I 'didn't want the nurses/midwives to slap me again. When I went to the hospital to deliver my second child, how the nurses beat me when I was delivering my baby and I 'wasn't pushing well"( IDI,multipara 3 woman)..*

Also, the participants reported that another poor conduct of skilled birth attendants that made them deliver at home was that the nurses and midwives neglected them. One participant indicated that she was very close to delivery, only to be told that the midwife has left the ward and went to the market to do shopping.

*"After the nurse attended to me and asked that I should go and be walking around, she left the room and went to the market to buy her things, and there was no one in the room except other three women who came and delivered and had not been discharged. When she left, I realised the baby was coming so I called one of the women who had delivered and was in the room with me to come and assist me, so the three of them left their children and quickly came to me and realised that the head of the baby was already out"( IDI,gravida 1 para 1 woman) .*

Furthermore, the participants indicated that one of the poor conducts of skilled birth attendants that made them give birth at home was the delay in rendering services to women any time they visited the health care facility. The participants narrated that sometimes when a woman is in labour and visits the health facility for delivery especially on weekends, they ‘don’t find the skilled birth attendants on duty. If this happens at night, they would be sleeping in their houses. And when they are called to attend to a labouring woman, it would take time before responding to the call as indicated by a participant below:

*"Hmm..., the last time I went to deliver at the health centre, when Joe went to call the midwife from her house, she delayed a bit before coming and yet I was in pain. But that is usual them. I mean the nurses will always delay when you get to the health facility to deliver. Sometimes, if it is a weekend, they will not even come to work unless you go to call them from their homes. So, why would you waste your time going there to deliver when labour starts in the night or during weekend" (IDI,gravida 2 para 1 woman).*

Also, the participants reported a lack of privacy and confidentiality in the provision of care during childbirth by skilled birth attendants as another poor quality of service. A participant expressed this:

*".... I was embarrassed when I realised people could see me from outside when I was in labour. The room where I delivered was very small, and some of the louvre blades were broken. Due to the heat in the room, they normally fold and tie-up the curtains to provide fresh air in the room. However, people who walk around the hospital can easily see you through the window. Sometimes, people walking outside usually see women in labour in the delivery room, and this is very embarrassing"(IDI,multipara 3 woman)..*

Participants indicated that some of the skilled birth attendants sometimes abandoned women under their care and go out of the health facility to do their personal things. As one of the participants indicated that she delivered by herself without any assistance of a skilled birth attendant in the health facility because the nurse who was on duty in the night left her and went home.

*"As for my case, it was serious. It was God who saved my life because when I went to deliver my second child at the hospital, the nurse who was on duty in the night left me and went home and the baby was coming, so I had to shout for my mother who was outside the ward to run and assist me. By the time my mother could get to the ward, the baby was already out and was on the floor" (IDI,gravida 1 para 1 woman).*

One of the participants indicated that when she was in labour and went to the nearest clinic to deliver, she got there and was told the midwife on duty had locked up the maternity ward and went away*.*

*".....We walked from my community to the Health Centre. When we got to the health centre, the midwife was not at post. So, I went back home and was assisted by a TBA to give birth"(IDI,gravida2 para2 woman).*

Another important factor expressed by some of the participants as having accounted for their decision to deliver at home was the environmental conditions at the health facilities. The participants indicated they were always expecting the maternity environment in the health facilities to be neat and secured like their various home environments but that was not always the case. One of the participants described the health facility environment as:

*"I think the labour room did not smell good. I realised when I entered the delivery room to deliver, somebody had just given birth and they 'didn't clean the room well before I entered so there was still visible blood on the table. As for the bath room, it was full of blood and the toilet was only one so people would defecate everywhere and you 'couldn't even breathe when you entered there" (IDI,multipara 3 woman).*

Another thing that created poor quality of care that forced them to deliver at home was lack of basic infrastructure and equipment such as beds and mattresses in most of the health facilities in the district. There were some of the facilities that women were seen lying on the bare floor because all the beds were occupied. One participant had this to say:

*"As for the hospital environment, I think the room where women who deliver stayed was just too small and so sometimes, it was just too crowded, and the windows were just too small and anytime there were lights out it was a big problem. Some of the mosquito nets were torn and so the mosquitoes were just disturbing us"(IDI,gravida 2 para 1 woman).*

Other participants also reported that some health facilities lacked supplies and equipment to conduct safe delivery. Participants indicated that there were times in some health facilities, where some drugs and other consumables such as disposable gloves were not available. All these make accessing health care in the health facilities the last option for women to consider. This was the comment from a participant:

*"……As for the health facility, they are always lacking one thing or the other. Either, they have run out of drugs or intravenous fluids or gloves. How can you go to a health facility to deliver when there are no drugs there"? (IDI,multipara 2 woman).*

#### Theme 2: women preferred home delivery because traditional birth attendants gave better care

The participants reported that they preferred home delivery because they perceived traditional birth attendants gave better care than the services that were rendered at the healthcare facilities by skilled birth attendants. Perception of women regarding the quality of skilled birth care as indicated previously, influence ‘women’s skilled birth care seeking behaviour. Participants semphasised the close bond they felt with TBAs, due to their status in the community and their trustworthiness. Some participants believed that they received high quality care from TBAs and believed that TBAs played a more supportive role. These descriptions were captured in the quote below:

*"If you deliver at home by the assistance a TBA or any relative, they will treat you very well. They will praise you and support you to go through the delivery process without feeling any pain. But in the hospital, the nurses will just be behaving as if they 'don't want you to come to them to deliver. They 'wouldn't help you, yet they will still insult you in addition. Their behaviour scares most women away from delivering in the health facilities" (IDI,multipara 2 woman).*

According to the participants, another reason that made them to prefer home delivery to skilled birth attendants was the availability or the opportunity for family members to be with the birthing woman when delivery assistance is being sought from a TBA. However, in health care facilities, family members are not permitted to enter the delivery room to offer support to the labouring woman. This was captured in the quote below:

*"When I delivered this my current baby at home, the way they treated me, I 'didn't even feel the pain during the delivery. My mother and other women were with me and they were singing and others were massaging my back and praising me throughout till I delivered" ( IDI,gravida2 para2 woman).*

The participants reported that the flexible choice of birthing position was also a factor that made them prefer home delivery with the assistance of TBAs to skilled birth care attendants. Most of the participants reported that unlike the healthcare facilities where women are compelled to adopt only the supine position in giving birth, the TBAs are flexible and a woman could adopt any position she felt comfortable with, provided it would not harm the mother and the unborn child. A participant expressed her view in the following statement:

*"One thing I liked about the TBA was that she asked me to sit on a stool and when I realised that I was not comfortable sitting and I told her about it she asked me to squat and see whether that was comfortable for me and I did. She assisted me to squat, I was in that position till I delivered my baby"( IDI,gravida1 para1 woman).*

Furthermore, the participants reported that they preferred home delivery because TBAs allowed family members and other relatives to prepare the traditional food, which according to the women are nutritious and good food for a newly delivered woman. A participant described the food as:

*"You know after delivery you are always weak and hungry so they need to give you something to eat; not just anything but something that will make you strong again. So, they give you """"zoomkom"""" warm millet water first to give you energy and to cleanse your mouth (meaning appetiser) and to help cleanse your stomach of all the dirt following the delivery, which will be followed with tuozaafi (local diet prepared from cereals), which will further boost your strength. The """"zoomkom"""" also helps you to produce more breast milk for your baby" (IDI,multipara 3 woman).*

The participants also reported that TBAs are very good at maintaining confidentiality of the birthing woman and the delivering process such as not exposing them to so many people during childbirth as compared to the health facilities. Participants indicated TBAs are very secretive about what happened during and after delivery and would not permit anybody who was not involved in the delivery process to watch them, unlike in the health facility, where they could allow many people including students to watch their nakedness. A participant noted:

*"There is privacy when you deliver at home. As for the hospital, there is no privacy. The last time I delivered my second child at the District Hospital, the nurses who were in the delivery room were more than five. Sometimes in the presence of all these people, they would just be insulting and shouting at you"(IDI,multipara 2 woman).*

#### Theme 3: women delivered at home due to financial constraints

Some of the participants indicated that they delivered at home due to financial constraints. The participants explained that although they knew about the importance of delivering at a health facility, and probably would have wished to deliver in a health facility, due to financial barriers of seeking care such as money to pay for transportation and other indirect costs involved in seeking skilled birth care, they were unable to utilise skilled birth care during childbirth. Participants expressed their concerns in the following quotes:

*"Most of us here deliver at home because of the poverty situation in this village. Majority of us here are not working and our husbands are not equally doing anything meaningful. So, if you 'don't have money, you 'can't go and hire a car to transport you to the health facility and to pay for other costs associated with delivery"(gravida3 para1 woman3).*

*"…I delivered at home because I 'didn't have money to pay for the cost of delivery, for transport and other things"( IDI,Gravida2 Para2 woman).*

The participants in this study indicated that apart from the lack of money to pay for transportation, paying for the cost of prescribed drugs that were not covered by the free maternal health care policy as well as buying food to sustain themselves and their caretakers during the period of stay in the health facility was another reason why women delivered at home. The descriptions of women regarding financial constraints are captured in the quote below:

*"As for the women in Amanga here, our problem is not about going to deliver in the health facility. Our main problem is how to get money to pay for drugs that are not given to us and the many things the skilled birth attendants would require of us to buy before we go to deliver in a health facility" (IDI,multipara2 woman).*

Another issue that was raised by participants that made them prefer home delivery during the data collection was that they did not have money to buy a delivery pack required by all women as part of their delivery plan. Participants noted that although most of the delivery services were covered under the free maternal healthcare policy, women still needed money to buy the delivery pack. The participants explained that because of their inability to buy the delivery pack, they chose to deliver at home to avoid embarrassment from skilled birth care providers.

*"We were told to buy pampers, sanitary pads, rubber (referring to mackintosh), soap, and Dettol (a kind of antiseptic). I 'can't remember all the things we were asked to buy but I think these were some of them and I 'couldn't buy any of them because they were very expensive and because I was not able to buy them I decided to deliver at home" (IDI,multipara 3 woman).*

Moreover, some of the participants reported that they did not get financial support from their husbands during their childbirth. They indicated that it was always difficult for them to get money from their husbands to buy the delivery set because according to the participants, the husbands either did not have money or ‘didn’t see the delivery set necessary. This was what one woman said:

*" Yes, I told my husband about those things (the delivery set) and he said he 'didn't have money. They usually do not see those things as important, so they 'wouldn't mind you when you are talking to them about those things. Sometimes, we also understand them because they really 'don't have money. Apart from the petty farming they do during the raining season which cannot even feed us well, they 'don't do anything that can earn any income"( IDI,multipara 4 woman)."*

#### Theme 4: women lacked access to healthcare facilities

The availability and accessibility of health facilities play an important role in the utilisation of skilled birth care in developing countries like Ghana. According to the participants of this study, issues such as long distance to health facilities, lack of transportation, inadequate skilled birth attendants in health facilities were some of the factors that made them lack access to healthcare facilities during childbirth. Childbirth services were geographically inaccessible to most of the population, as captured in the quote below:

*"he distance to the health facility is a major problem for women here in Amanga. Because there is no health facility here, people would have to travel as far as to Namoo to access skilled birth care. The long distances to health facilities discourage women from accessing skilled birth care services in Amanga"(IDI,gravida3 para2 woman).*

The participants reported that the problem of long distance was exacerbated by poor roads, rivers and valleys separating some of the communities from the health facilities that provide skilled birth care. For instance, some of the participants indicated that one thing that made it even more difficult accessing health facilities in their community was the fact that women would have to cross two major rivers before they could get to a health centre to access care. As a result, some ended up delivering at the riverside as depicted below:

*"The rivers here in Amanga serve as a barrier to accessing care anytime it rains. I even delivered my second child by the riverside because it was in the rainy season and when I was in labour and got there, the river was full and we 'couldn't cross it, so I delivered by the riverside"( IDI, gravida2 para2 woman).*

Another problem of access to the healthcare facilities as reported by most participants in this study was lack of means of transport to healthcare facilities. The most common means of transport, usually bicycles, motorbikes or sometimes tricycles which were often the only alternatives were risky and sometimes culturally unacceptable as illustrated below:

*"What really informed my decision to deliver at home was the fact that the labour started in the night and I 'didn't have any means of transport to get to the clinic to deliver"(IDI*,*multipara3 woman).*

## Discussion

This study sought to explore and describe why women in rural northern Ghana give birth at home without utilising the skilled birth attendants available in health facilities. The findings of this study brought to light an understanding that accounted for women giving birth at home in rural areas in Ghana. The results of this current study showed that one of the reasons why participants preferred to give birth at home despite the importance of skilled birth attendance was poor quality of care and attitude of skilled birth attendants. It was reported in this study that poor quality of care and attitude of skilled birth attendants such as using harsh words on women during childbirth, subjecting labouring women to physical abuse, verbal abuse, neglect, discrimination, and denial of traditional practices during labour and delivery were exhibited by midwives. Participants indicated that because they had ever experienced poor conduct of skilled birth attendants during childbirth, and neglected by nurses and midwives in their previous child birth, they were deterred them from going to give birth in such facilities again. A ‘woman’s perceptions of the attitude of health care providers due to her previous experience of care can affect her future decision to seek care, especially during childbirth. In common with the current study findings, a recent study conducted in rural northern Ghana, revealed that women reported that midwives and nurses shouted at women, insulted them, and spoke harshly to them [21]. Similarly, a study in Tanzania reported that women described the fear of arriving at a facility and being ignored or being verbally abused by skilled birth providers as the major reason that accounted for women giving birth at home utilising the birth care provided by TBAs in Tanzania [22]. It is important to note that poor conduct of skilled birth attendants occur in Ghana and other developing countries, deterring women from going to give birth in a health facility [23].

Moreover, the finding of this study showed that, one of the reasons that accounted for home births was women’s perception that traditional birth attendants gave better care than the birth care provided by skilled birth attendants at the healthcare facilities in rural areas. Participants expressed that care provided by TBAs were adequate and TBAs approach to childbirth fulfils the expectations of the labouring mothers and their immediate families in a way that the modern health system does not. Participants in this current study semphasised the close bond they felt with TBAs, due to their status in the community and their trustworthiness. Previous studies have consistently reported women perception of quality of care provided by TBAs as a major setback to achieving the goal of reducing maternal mortality in rural communities in developing countries such as Ghana [24–28]. For instance, a recent study conducted in Ghana reported that women continued to give birth at home because of the perception that TBAs give better care as compared to the poor quality of care at health facilities in the rural areas [29]. These findings highlight the importance of collaborative maternity care between skilled birth attendants and TBAs in order to meet the needs of labouring women and as well reduce maternal mortality in rural areas.

Furthermore, financial constraint was largely cited by majority of the participants in this current study as a major reason that motivated them to opt for home births. The participants explained that although they knew about the importance of delivering at a health facility, and probably would have wished to deliver in a health facility, however, financial barriers (such as money to pay for transportation and other indirect costs involved in seeking skilled birth care) involved in seeking care in the established health facilities, they were unable to utilise skilled birth care during childbirth. Although, maternity care is free in Ghana for all women following the introduction of the Free Maternal Policy by the government of Ghana in 2008, women during childbirth still incur indirect costs that are not taken care of by the policy. The purpose of the policy was to eliminate financial barriers that could hinder uptake of maternal health services by women especially the poor, thus increasing skilled attendance at delivery. However, because of the indirect cost (expenses incurred not on treatment) incurred by women such as paying for transportation to get to the facility which is not covered under the free maternal care policy, and these costs could prove to be expensive and might have deterred some women from utilising facility-based delivery services. A study in Ghana states that in the rural communities where much of the population is extremely poor and where most families rely on subsistence agriculture for survival, despite the free maternal policy, financial challenges such as paying for transportation to health facility still impede facility-based deliveries [15]. Another study states that even in settings where direct delivery costs were subsidised, families were expected to pay for transportation to the facility and still buy drugs, medical supplies (i.e. gloves, needles, gauze), blood for transfusions, laboratory services, food during the hospital stay, bribes to health service providers, and laundry services which are usually expensive for the poor woman in the deprived community [30]. Sometimes, the above-mentioned additional costs often came as a surprise to women after they attended the facility, which may impact their future choice of delivery location.

Also, the lack of access to maternal healthcare facilities by labouring women was reported in this current study as a major reason associated with home birth. The availability and accessibility of health facilities play an important role in the utilisation of skilled birth care in developing countries like Ghana. According to the participants of this study, issues such as long distance to health facilities, lack of transportation, inadequate skilled birth attendants in health facilities were some of the factors that contributed to their of lack access to healthcare facilities during childbirth. Bongo district is one of the deprived districts in Ghana with extremely poor infrastructure, inadequate health facilities with the required qualified midwives and nurses and it is compounded with deplorable roads. The district has only one hospital and five health centres and clinics with few qualified midwives that provide skilled birth care. The ‘researchers’ observation confirmed that some women in some communities in the district travel long distance to access skilled birth care. Poor roads, rivers and valley in some of the communities meant that during the rainy season or in case of obstetric emergencies, pregnant women could only reach health facilities if they were carried by men, which could be risky and cause delay in seeking care. In consistent with the finding of this study, lack of access to essential maternal healthcare services has been identified as the main underlying causes of maternal deaths in Sub-Saharan Africa and other developing countries [31]. It was reported in a previous study conducted in rural northern Ghana that sometimes women in labour would attempt to risk their lives by going through the rains to a health centre to give birth only to realise that the rivers were full to the brim making it impossible for them to cross over [27]. The researchers in this current study observed that almost all the roads in the study communities were not tarred and they were not suitable for transport especially during the rainy season. Most villages are connected to the health facilities only by footpaths. The flat terrain coupled with rivers, poor drainage of water predisposes the land to perennial floods which last several months.

## Conclusion

The findings of this study brought has to light the barriers to the utilisation of birth care services provided by skilled birth attendants in rural northern Ghana. The study also revealed that women in rural and deprived areas like the Bongo district of Ghana lack access to skilled birth care due to unavailability of healthcare facilities, absence of skilled birth attendants during night and weekends as well as geographical barriers such as bad road networks, long distances to health facilities and lack of means of transport. Management of healthcare facilities in rural communities should facilitate the implementation of a supportive supervision in the maternity units to improve the quality of care and attitude delivered by skilled birth attendants in maternity care in rural communities.

### Implication for nursing and midwifery practice

- Management of healthcare facilities should facilitate the implementation of a supportive supervision in the maternity units to improve the quality of care and attitude delivered by skilled birth attendants in maternity care in rural communities.
- Management of healthcare facilities should facilitate the implementation of capacity training programmes for skilled birth attendants to improve their skills and competency
- Management of healthcare facilities should facilitate the implementation of health education programmes for pregnant women to create ‘women’s awareness about the importance of skilled birth attendance and birth preparedness.

## Supplementary information

**Additional file 1.** Interview guide

## Data Availability

The transcripts from which this manuscript was developed are available on request from the corresponding author.

## Abbreviations

TBA: Traditional Birth Attendant
GSS: Ghana Statistical Service
CHPS: Community Health-based planning Services
CKIs: Community Key Informants
GDHS: Ghana Demographic and Health Survey
