# Supplementary material for: Exploring the reasons why women prefer to give birth at home in rural northern Ghana: a qualitative study
Source: BMC Pregnancy Childbirth. 2020 Aug 28;20:500. doi: 10.1186/s12884-020-03198-y (PMC7456369; doi:10.1186/s12884-020-03198-y)
Supplement: Supplementary file 1 — Additional file 1. Interview guide [file 12884_2020_3198_MOESM1_ESM.docx]

Supplementary file

**INTERVIEW GUIDE**

1. Please tell me something about yourself
2. Please tell me all about your experiences during the labour and birth of this current baby?
3. Please tell me the reason (s) why gave birth at home without utilising the services of skilled birth attendants such as doctors, nurses and midwives?
4. Please tell me your recommendations to improve skilled birth care services at the health facilities in your community.
